# Association of Past and Future Paid Medical Malpractice Claims

**DOI:** 10.1001/jamahealthforum.2022.5436

**Published:** 2023-02-10

**Authors:** David A. Hyman, Joshua Lerner, David J. Magid, Bernard Black

**Affiliations:** 1 Georgetown University Law Center, Washington, DC; 2 NORC at the University of Chicago, Chicago, Illinois; 3 School of Public Health, University of Colorado, Lafayette; 4 Kellogg School of Management and Pritzker School of Law, Northwestern University, Chicago, Illinois

## Abstract

**Importance:**

Many physicians believe that most medical malpractice claims are random events. This study assessed the association of prior paid claims (including a single prior claim) with future paid claims; whether public disclosure of prior paid claims affects future paid claims; and whether the association of prior and future paid claims decayed over time.

**Objective:**

To examine the association of 1 or more prior paid medical malpractice claims with future paid claims.

**Design, Setting, and Participants:**

This study assessed the association between prior paid claims (including a single prior claim) with future claims; whether public disclosure of prior claims affects future paid claims; and whether the association of prior and future paid claims decayed over time. This retrospective case-control study included all 881 876 licensed physicians in the US. All data analysis took place between July, 2018 and January, 2023.

**Exposure:**

Paid medical malpractice claims.

**Main Outcome and Measures:**

Association between a prior paid medical malpractice claim and likelihood of a paid claim in a future period, compared with simulated results expected if paid claims are random events. Using the same outcomes, we also assessed whether public disclosure of paid claims affects future paid claim rates.

**Results:**

This study included all 881 876 physicians licensed to practice in the US at the time of the study. Overall, 3.3% of the 841 961 physicians with 0 paid claims in the prior period had 1 or more claims in the future period vs 12.4% of the 34 512 physicians with 1 paid claim in the prior period; 22.4% of the 4189 physicians with 2 paid claims in the prior period; and 37% of the 1214 physicians with 3 paid claims in the prior period. The association between prior claims and future claims was similar for high-medical-malpractice-risk and lower-risk specialties; 1 prior-period claim was associated with a 3.1 times higher likelihood of a future-period claim for high-risk specialties (95% CI, 2.8-3.4) vs a 4.2 times higher likelihood for lower-risk specialties (95% CI, 3.8-4.6). The predictive power of a prior paid claim for future claims declined gradually as the time since the prior claim increased, for prior or future periods up to 10 years. Public disclosure did not affect the association between prior and future paid claims.

**Conclusions and Relevance:**

In this study of paid medical malpractice claims for all US physicians, a single prior paid claim was associated with substantial, long-lived higher future claim risk, independent of whether a physician was practicing in a high- or low-risk specialty, or whether a state publicly disclosed paid claims. Timely, noncoercive intervention, including education, has the potential to reduce future claims.

## Key Points

**Question:**

Do prior paid medical malpractice claims predict future paid claims?

**Findings:**

In this retrospective, case-control study including all 881 876 physicians licensed to practice in the US at the time of the study, physicians with 1 paid claim (regardless of specialty) were almost 4 times more likely to have 1 or more paid claims in the next 5 years compared with physicians with no prior paid claims. The likelihood of future claims rose monotonically with the number of prior claims and was unaffected by whether paid claims were publicly disclosed.

**Meaning:**

The findings of this study suggest that paid medical malpractice claims are not random events; timely noncoercive intervention has the potential to reduce future claims.

## Teaser

Using data from the National Practitioner Data Bank, this case-control study examines the association between a prior paid medical malpractice claim and the likelihood of a paid claim in a future period, compared with simulated results expected if paid claims are random events.

## Introduction

It has long been known that some physicians are prone to multiple medical malpractice claims. These claim-prone physicians have attracted considerable public concern.^1,2^ One concern is whether hospitals are too liberal in granting privileges, and state medical boards are too willing to grant or renew licenses for physicians with multiple paid claims.

Physicians typically argue that a medical malpractice claim, especially a single claim, is often a random event, reflecting bad luck rather than lack of skill. In this view, past claim history would not be associated with future paid claims, except to the extent that plaintiffs’ lawyers are more likely to bring claims against physicians with known paid claims (ie, a “blood in the water” effect). Prior research shows that, controlling for specialty, physicians with multiple prior paid claims face higher future claim risk than physicians with a single prior paid claim.^3^ A single-state study using 1980s data assessed whether physicians with 1 or more prior claims faced higher future claim risk than physicians with no prior claims.^4^ Our recent study using national data from 2006 to 2016 examined the same issue.^5^ But, to our knowledge, no prior study has assessed whether public disclosure of paid claims affects future claim rates. Similarly, we know of no prior study that has evaluated the relative association of future claims with recent vs more distant prior paid claims.

This study used data on paid medical malpractice claims for all physicians in the US to assess the association between experiencing 1 or more paid claims in a prior 5-year period (2009-2013) and the risk of 1 or more future paid claims in the next 5 years (2014-2018). This study also compared observed claim rates with those that would be expected if paid claims were random events, that arrived at rates that depend on state, but are independent of other physician characteristics. The study also assessed whether public disclosure of past paid claims was associated with future paid claim rates, and how the strength of the association between prior and future paid claims varied with the time since a prior paid claim occurred. Finally, the study used data from Illinois to assess whether the association of prior paid claims and future paid claims was different for physicians in high– vs low–malpractice-risk specialties.

## Methods

The study followed the Strengthening the Reporting of Observational Studies in Epidemiology (STROBE) reporting guidelines. The principal data source for this study is the National Practitioner Data Bank (NPDB), a national repository of all paid medical malpractice claims since 1992 involving individual health care professionals; this study focused on physicians with an MD. Each physician received an anonymized, time-consistent identifier.

Counts of active practicing nonfederal physicians (below, “active physicians”) in the 51 states (50 states plus DC) were obtained from the Area Health Resource File. Data on when physicians enter or leave active practice is not available, so the study assumes no entry or exit when evaluating the association of prior with future paid claims.

This study compared the actual and simulated probabilities that a physician in a given state, with a specified number of prior paid claims in a prior period (for example, 1 claim in the past 5 years) will have 1 or more paid claims in a subsequent future period (for example, 1 or more claims in the next 5 years). The principal results used a 5-year prior period from 2009 to 2013, and a 5-year future period from 2014 to 2018. The study grouped physicians based on the number of prior-period paid claims (0, 1, 2, or ≥3 claims), and measured the number of members of each group who experience 0, 1, 2, or 3 or more future-period paid claims.

The simulation of the paid claim counts that would be expected with random claim arrival at state-specific rates was derived by computing observed state-specific annual risk of a paid claim, defined as:

f_state_ = (average No. of claims in the state over 2009-2018)/(average number of physicians in the state over 2014 to 2016 [first 3 years of future period]).

State-specific annual claim risk was used for each physician because of wide variation in state-level claim frequency (eTable 1 in Supplement 1). The state-specific risks were used to simulate how many claims each physician in each state would receive in the future period if claims arrived randomly at state-specific rates. In each simulation, claims were randomly assigned to physicians at the appropriate state-specific rate. For example, if there were *Y* annual claims in state *X*, which had *n* practicing physicians, claims were assigned at random to the physicians in that state, so that the expected number of paid claims for all physicians was *Y*. The number of physicians nationally who received 0, 1, 2, or 3 or more pseudo claims over the future period was determined and then rounded up to the nearest whole number. This simulation was run 10 000 times, and the counts were averaged and again rounded up to the nearest whole number. The simulation runs were also used to measure 95% CIs around the average counts. See eMethods in Supplement 2 for additional simulation details.

To evaluate the relative association of more distant vs more recent prior claims with future claims, the duration of both the prior and future periods was varied from 1 to 10 years. To assess whether public disclosure of prior paid claims was associated with future paid claims, future paid claim rates for the 19 states that publicly disclose which physicians have paid malpractice claims were compared with future paid claim rates in the remaining states, which do not disclose this information.

Because the NPDB data set does not include physician specialty, the study used data from Illinois from 1990 to 2016 (see Hyman et al, 2021, for data set details^6^). Illinois physicians were divided into specialties with high medical malpractice risk (obstetrics and gynecology, general surgery, and all surgical specialties) vs lower-risk specialties (all others) based on specialty-specific risks of paid claims (eTable 2 in Supplement 1). The prior period was 2005 to 2009; the future period was 2010 to 2014. The analysis was otherwise similar to the national analysis and was run separately for each group.

## Results

Table 1 shows the actual number of paid claims during the study period, stratified by number of paid claims (0, 1, 2, or ≥3) during the prior period (the 5 years from 2009-2013). During the prior period approximately 96% of physicians had 0 claims, 3% had 1 claim, and less than 1% had 2 or more claims. There is a similar pattern during the 5-year future period (2014-2018): 97% of physicians had 0 claims, 3% had 1 claim, and less than 1% had 2 or more claims. eTable 3 and the eFigure in Supplement 1 provide additional summary statistics on paid claim rates.

**Table 1.** Summary Statistics and Risk for Future Paid Claims Given Prior Claim History

Prior period (2009-2013)		Future period (2014-2018)^a^						
Paid claims per physician	Physicians, No. (%)	Total paid claims	Physicians with paid claims, No.				Physicians with future claims, %	
			0	1	2	≥3	≥1	≥2
0	841 961 (95.92)	0	814 036	25 051	2370	504	3.32	0.34
1	34 512 (3.42)	34 512	30 236	3466	604	206	12.39	2.35
2	4189 (0.51)	8378	3250	669	189	81	22.42	6.45
≥3	1214 (0.15)	4669	765	274	83	92	36.98	14.42
Total	881 876 (100)	47 559	848 287	29 460	3246	883	3.81	0.47
Estimated with random arrival of paid claims (95% CI)			840 672 (834 510-846 866)	40 209 (34 118-46 240)	963 (819-1107)	16 (14-18)	4.78 (4.06-5.45)	0.12 (0.10-0.14)

^a^ The total actual claims in the future period was 39 691.

Table 1 shows that the risk of a future-period claim increased monotonically with the number of prior-period paid claims. For example, the proportion of physicians with 1 or more future-period claims was 3.3% for the 841 961 physicians with 0 prior-period claims, 12.4% for the 34 512 physicians with 1 prior claim, 22.4% for the 4189 physicians with 2 prior claims, and 37% for the 1214 physicians with 3 or more prior claims. Relative to physicians with no prior-period claims, the risk of a future-period claim was 3.7 times higher for physicians with 1 prior-period claim (95% CI, 3.3-4.4); 6.7 times higher for physicians with 2 prior-period claims (95% CI, 5.9-7.9); and 11.2 times higher for physicians with 3 or more prior-period claims (95% CI, 9.8-13.1). Similarly, the proportion of physicians with 2 or more future-period claims was 0.3% for the physicians with 0 prior-period claims, 2.4% for physicians with 1 prior paid claim, 6.5% for physicians with 2 prior paid claims, and 14.4% for physicians with 3 or more prior paid claims. Consistent patterns are found if one separates the 3 or more group into 3 vs 4 or more claims (eTable 4 in Supplement 1).

**Figure 1.**
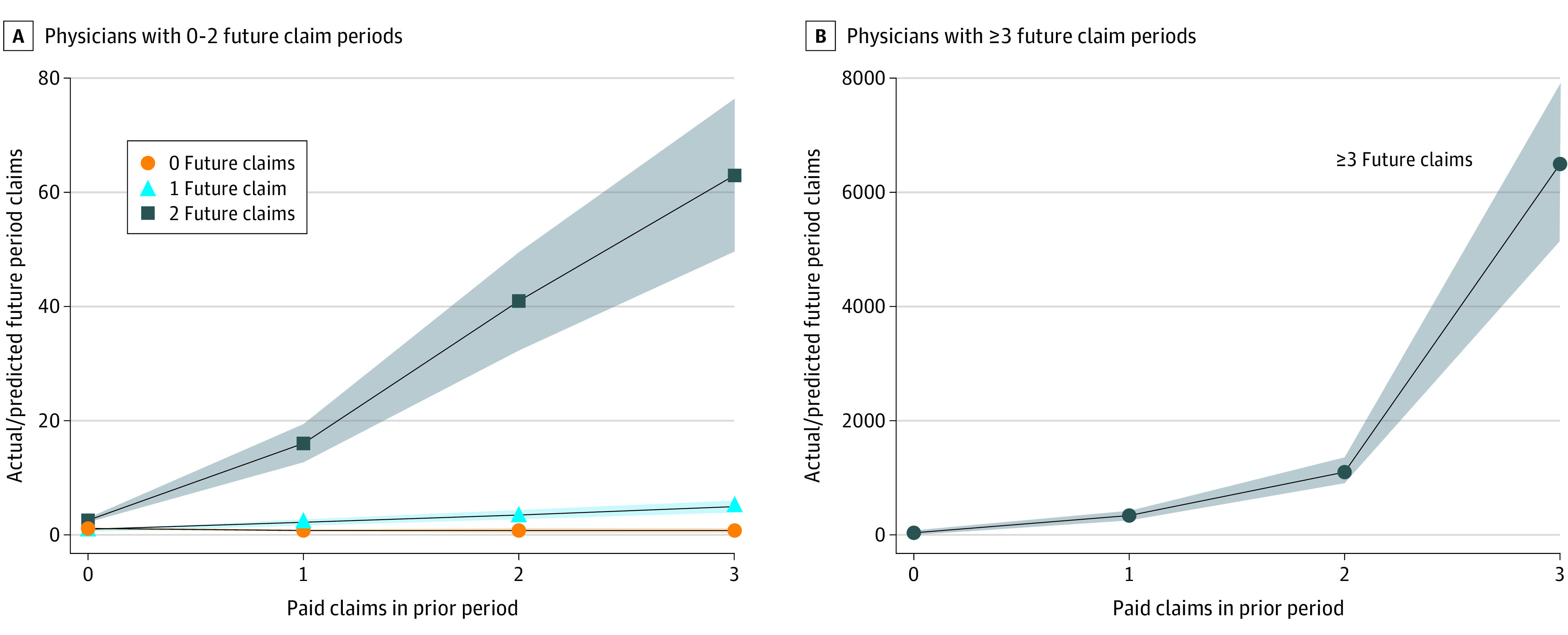
Ratio of Actual Future Period Claims to Number Expected With Random Claim Arrival Illustrated are the ratios of the actual likelihood of having a given number of future-period paid claims (based on Table 1) to the average likelihood for a physician to have the indicated numbers of future-period claims if claims arrived at the average state-specific rates observed for the prior period, but otherwise at random (independent of physician skill) and all physicians active during the prior period remained active in the future period. A, The ratio of actual/predicted future-period claims varied with number of prior claims for physicians with 0, 1, or 2 future-period claims, using a y-axis scale running from 0 to 80. B, The ratio of actual/predicted future-period claims for physicians having 3 or more future-period claims using a y-axis scale running from 0 to 8000. The shaded areas above and below each main line represent the 95% CIs for estimates. See eTable 4 (Panel D) in Supplement 1 for numerical values underlying this Figure.

Figure 1 illustrates, in a different way, how the likelihood of a future paid claim, or multiple future paid claims, varies with the number of prior-period claims. Figure 1 shows the ratio of actual future-period claims to the number expected from our simulation of random claim arrival. The likelihood of future claims rose with the number of prior-period claims, the actual-to-predicted ratios became large for physicians with multiple prior-period claims, multiple future- period claims, or both (see eTable 5 in Supplement 1 for the underlying data). Figure 1A shows these ratios for physicians with 0, 1, or 2 prior-period claims, using a y-axis scale running from 0 to 80. Physicians with 1 paid claim in the prior period were 16 times more likely to have 2 future-period claims (95% CI, 12.5-19.3), and physicians with 3 or more prior-period claims were 63 times more likely to have 2 future-period claims than would be the case with random claim arrival (95% CI, 50-76). Figure 1B shows ratios for physicians with 3 or more future-period claims, using a y-axis scale running from 0 to 8000. Physicians with 1 prior-period claim were 338 times more likely to have 3 future-period paid claims (95% CI, 267-409), and physicians with 3 prior-period claims were 6506 times more likely to have 3 future-period claims, relative to random claim arrival (95% CI, 5140-7872). These high ratios reflect the meaningful numbers of physicians who had these patterns, shown in Table 1, vs the low probabilities that these patterns would be observed if claims arrived at random.

In Table 2, the study uses a 10-year prior period, divided into a pre-preperiod (years −10 to −6) and a preperiod (years −5 to −1). Table 2 reflects the likelihood of a future-period paid claim for physicians with (1) 1 or more claims in both the pre-preperiod and the preperiod; (2) 1 or more claims in 1 period but not the other, and (3) physicians with no claims in either prior period. Physicians with paid claims in both prior periods had the highest risk (23.0%); physicians with 0 claims in both prior periods had the lowest risk (2.8%). For physicians with a paid claim in 1 of the prior periods, those with a more recent paid claim had a 12.2% risk of a future period claim, vs 8.4% for those with an older prior claim.

**Table 2.** Risk Ratios for Future-Period Claims, Using Both a Preperiod (years −5 through −1) and a Pre-Preperiod (years −10 to −6)^a^

Variable	Prior claims, No.	Preperiod (2009-2013)		Difference across columns, *P* value
		0	≥1	
Pre-preperiod (2004-2008)	0	23 935/841 961 (2.84)	3530/28 934 (12.20)	<.001
	≥1	3208/38 327 (8.37)	1477/6421 (23.01)	<.001
Difference across rows, *P* value		<.001	<.001	NA

^a^ The likelihood of having 1 or more paid claims in a future 5-year period based on whether the physician had (1) 0 claims in both the pre-preperiod (years −10 through −6) and the preperiod (years −5 through −1); (2) 0 pre-preperiod claims but 1 or more preperiod claims; (3) 1 or more pre-preperiod claims but 0 preperiod claims; or (4) 1 or more claims in both the pre-preperiods and periods. Each cell shows numerator (number of physicians in cell with a future-period paid claim) and denominator, and the percentage in parentheses. Denominator for 0 to 0 cell is from American Medical Association annual surveys, averaged over 2009 to 2013. Denominators for other cells are from the National Practitioner Data Bank. NA indicates not applicable.

In eTable 6 in Supplement 1, we varied the length of the future period from 1 to 9 years. Consistent with the difference in Table 2 between future claim rates for physicians with pre-preperiod vs preperiod paid claims, the association between prior-period and future-period paid claims gradually weakened as the length of the future period increased.

**Figure 2.**
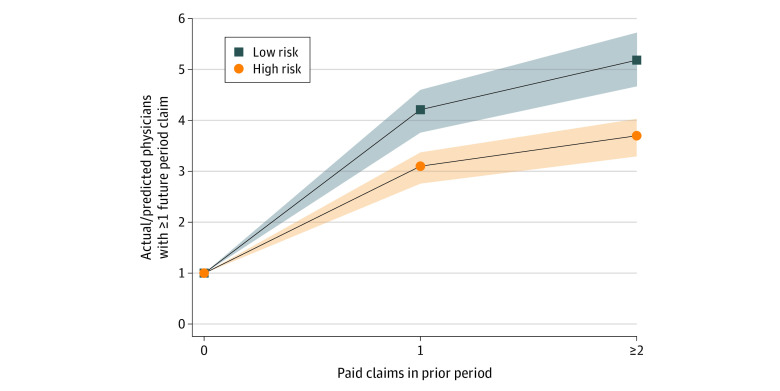
Relative Future-Period Paid Claim Risk for Illinois High-and Lower-risk Specialties Illustrated is the ratio of the actual likelihood that an Illinois physician will have a paid claim in the future period (2010-2014) to likelihood if claims arrived randomly at the average rate for all high-risk (lower-risk) specialties. The data set was paid medical malpractice claims for Illinois physicians over the period from 1990-2016. Predicted numbers are mean values from 10 000 simulations. Ratios are normalized to likelihood for physicians with 0 paid claims in the prior period (2005-2009). High-risk specialties include obstetrics and gynaecology, surgery (including surgery subspecialties), urology, and otolaryngology. Lower-risk specialties are all other specialties. Dashed or dotted lines above and below each main line represent the 95% CIs for estimates. See eTables 8 and 9 (Panel C) in Supplement 1 for numerical values underlying this Figure.

What about variation by specialty? Figure 2 uses Illinois data and shows the ratio of actual-to-predicted physicians with 1 or more future-period paid claims, based on the number of prior-period claims, separately for high-malpractice-risk and lower-risk specialties. eTables 2 and 7 in Supplement 1 provide Illinois and national data on specialty-specific risks. Physicians with 1 prior-period claim in lower-risk specialties had a 4.2 times higher risk of a future-period claim (95% CI, 3.8-4.6), compared with physicians in high-risk specialties who have a 3.1 times higher risk of a future-period claim (95% CI, 2.8-3.4). Physicians with 2 or more prior-period claims in lower-risk specialties had a 5.2 times higher risk of a future-period claim (95% CI, 4.7-5.7), compared with physicians in high-risk specialties who had a 3.7 times higher risk of a future-period claim (95% CI, 3.3-4.0). Thus, although absolute future-period claim risk was higher for high-malpractice-risk specialties, the relative increase in future claim risk for physicians with vs without prior-period claims was similar for high- and low-risk specialties.

**Figure 3.**
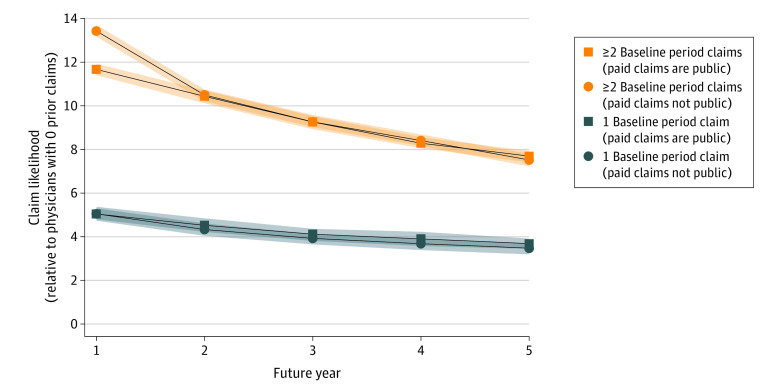
Effect of Public Disclosure of Paid Claims on Risk of Future Paid Claims The ratio of (1) the probability that a physician with the indicated number of prior-period paid claims will have 1 or more future-period paid claims to (2) this probability for physicians with 0 prior-period paid claims. The lines with squares show ratios for states where paid medical malpractice claims are publicly reported; the lines with circles show ratios for states where paid claims are not publicly reported. The shaded areas above and below each main line represent the 95% CIs for estimates. See eTable 10 in Supplement 1 for numerical values.

The study also assessed whether physicians were more likely to face future claims because of public disclosure of prior paid claims. The likelihood of future paid claims, given similar prior-period history, was compared for the 19 states in which paid claims are publicly disclosed vs the remaining states, where prior-claim records are generally not available. Figure 3 shows that public disclosure of prior paid claims had no significant effect on the likelihood that physicians would experience future-period paid claims. The bottom lines (lines with squares for states in which paid claims are publicly reported; lines with circles for the other states) show the ratio of the likelihood that a physician with 1 prior-period paid claim would have 1 or more future-period paid claims vs this probability for physicians with no prior-period claims. The top lines are similar, but the numerator of each ratio is the number of physicians with 2 or more prior-period paid claims. If public reporting of paid claims made physicians more likely to experience additional paid claims, the dashed lines would be above the solid lines. This is not observed.

## Discussion

Physicians with even a single paid medical malpractice claim in a prior period were shown to have a greatly elevated risk of having additional paid claims during a future period. With 5-year prior and future periods, a single paid claim in the prior period was associated with a roughly 4 times higher likelihood of a future-period paid claim, relative to the likelihood for physicians with no prior-period claims. The elevation of risk was similar for both high-risk and lower-risk specialties. The greater the number of prior-period paid claims, the greater the likelihood of having a paid claim over any given future period, as well as the expected number of future-period claims. This pattern was not affected by whether plaintiffs’ lawyers had access to information about physicians’ past paid claims.

The increased future-period risk, relative to the risk physicians would face if claims were random events, was much higher for physicians who had multiple paid claims in both the prior and future periods. For the 1214 physicians with 3 or more prior-period claims, 83 had 2 future-period claims, and 92 had 3 or more future-period claims. Yet if claims arrived at random, there should be no physicians in either group (the simulated number was less than 0.1 for 2 future-period claims, and less than 0.02 for 3 or more future-period claims).

Physicians with 1 or more claims in both a pre-preperiod (years −10 to −6) and a preperiod (years −5 to −1) had higher risk of a future-period claim than those with a prior claim in 1 but not both periods, and much higher risk than those with no prior claim in either period. The degree of association between past and future claims fell gradually as the time since the prior-period claim increased.

Some factors that are associated with future claim risk are ones that hospitals and medical boards cannot act on (ie, age, gender, and specialty).^3,4,5,7,8,9,10,11,12,13,14,15^ In addition, prior research suggests^10^ that some physicians are claim-prone because of issues with their communication skills/bedside manner, as opposed to technical skill. We could not distinguish between these 2 sources of claim risk with our data, but note that our data are limited to claims that are paid, not merely initiated. Future work will be needed to disaggregate the contribution of each of these elements to claim-proneness.

Paid claims are an imperfect signal of low-quality care. Still, we offer evidence that even 1 claim provides an important signal, and that multiple claims provide a strong signal. This signal can likely be strengthened by combining information on paid claims with data on unpaid medical malpractice claims, specialty, and disciplinary sanctions imposed by state medical boards, loss of hospital privileges, and other adverse events.

Can our findings of a strong association between a single prior-period paid claim and future claim risk be reconciled with the common physician view that many malpractice claims are random events and that someone with a single paid claim was probably just unlucky? Prior work indicates that only a small percentage of medical errors lead to claims, but a substantial majority of paid claims reflect probable negligence.^15,16^ We provide a simple formal model in the eAppendix in Supplement 1, which builds on these background facts, in which the positive predictive value (PPV) of a paid claim (the likelihood that a paid claim reflects actual negligence) depends on physician skill, and could be low for high-skill physicians. Our results for the association between a prior and a future paid claim reflect the average signal conveyed by prior claim history, across both high-skill and low-skill physicians.

If future claim risk is reliably associated with past claims, one should consider interventions designed to reduce future claim risk. Even physicians with a single paid claim could benefit from efforts aimed at reducing future claim risk, despite the potential for the paid claim to be a false positive. Intervention following a single paid claim should be voluntary, and could be as simple as offering continuing medical education opportunities focusing on error avoidance and posterror communication with patients.

For physicians with 2 or more paid claims, especially within a limited time period, stronger interventions should be considered. Possible steps for physicians with 3 or more paid claims (or perhaps 2 or more in a low-risk specialty) could include closer supervision; counseling to improve their communication skills; refresher training; and perhaps encouragement to move to nonclinical practice. Outlier paid claim records could justify investigation of practice patterns, to assess whether license suspension or revocation is appropriate. The extent of interventions, and whether they are voluntary or mandatory, should reflect the number and recency of prior paid claims. Implemented properly, such graduated strategies have the potential to reduce future paid claims and patient harm.

### Limitations

This study has some limitations. Data on how many patients each physician sees were not available. But in our analysis, each physician served, in effect, as his or her own control. This approach relies on physician practice patterns being reasonably stable over time, including volume of patients seen and changes in patient mix. We are not aware of any data indicating that patient volume is likely to fluctuate greatly during the period when a physician is in active practice. Patient mix could change if a physician moves from one practice setting to another.

We cannot observe whether some physicians are willing to treat riskier patients and thus face a higher risk of adverse outcomes and thus medical malpractice claims. We did not observe unpaid claims. Some claims may not be reported to the NPDB, although in prior work^6^ we found close correspondence between claims reported to the NPDB and claims reported to the Illinois Department of Insurance and to the Illinois Department of Professional and Financial Responsibility.

We did not observe physician entry and exit, and our analysis assumed no entry or exit of physicians between the prior and future periods. There is evidence that physicians with prior paid claims leave medical practice at somewhat higher rates than other physicians.^14,17^ These higher exit rates would bias downward the tendency we found for physicians with prior paid claims to have higher future claim risk than physicians without prior paid claims.

For physician specialty, we only had data from Illinois, but Studdert et al^3^ found similar results for physicians with 1 or more paid claims using national data.

## Conclusions

In this case-control study of the association between prior-period paid malpractice claims and future-period claim risk, having even 1 prior-period paid claim was associated with a roughly 4-fold higher risk of a future-period paid claim, and even more elevated risk of having multiple future-period claims. This study also found that having multiple prior claims predicted a very large increase in the likelihood of multiple future-period claims, relative to either the null hypothesis of random claim arrival, or the actual claims experienced by physicians with no prior-period claims. The association between prior-period claims and future-period claim risk is similar for high-malpractice-risk and lower-risk specialties. We also find no evidence of a blood in the water effect, in which public disclosure of prior paid claims increases future-claim risk. Some paid claims are false-positive claims, but taken as a whole, paid claims convey a strong signal of future risk. The policy challenge is how to use this information to reduce future medical malpractice claims and patient harm without overreacting to the signal conveyed by a single paid claim.

## Notes

Supplement 1. eAppendix. Simple Model: Medical Malpractice Claims as an Imperfect Proxy for Negligent Care Simulation Methodology eTable 1. Risk of a Paid Claim by State eTable 2. Specialty-Specific Risk of Paid Claims: Illinois Data eTable 3. Panel A. NPDB Summary statistics by claim year eTable 3. Panel B. Percentage of Physicians with Paid Claims Over Different Time Periods eTable 4. Summary Statistics, and Risk for Future-Period Claims, Given Prior-Period Claim History eTable 5. Ratio of Actual to Predicted Future Claims eTable 6. Risk Ratios for Future Claims, for Varying Periods eTable 7. Paid Claim Risk by Specialty (Risk Level from Jena et al, 2011) eTable 8. Risk for Future Claims for Illinois high-risk specialties eTable 9. Risk for Future Claims for Illinois Lower-Risk Specialties eTable 10. Effect of Public Disclosure of Paid Claims on Future Claim Risk eFigure 1. Total Paid Claims per 1000 Active Physicians from 1992-2016

Supplement 2. Data Sharing Statement
