# Supplementary material for: Association of Past and Future Paid Medical Malpractice Claims
Source: JAMA Health Forum. 2023 Feb 10;4(2):e225436. doi: 10.1001/jamahealthforum.2022.5436 (PMC9918873; doi:10.1001/jamahealthforum.2022.5436)
Supplement: Supplement 1. — eAppendix. Simple Model: Medical Malpractice Claims as an Imperfect Proxy for Negligent Care Simulation Methodology eTable 1. Risk of a Paid Claim by State eTable 2. Specialty-Specific Risk of Paid Claims: Illinois Data eTable 3. Panel A. NPDB Summary statistics by claim year eTable 3. Panel B. Percentage of Physicians with Paid Claims Over Different Time Periods eTable 4. Summary Statistics, and Risk for Future-Period Claims, Given Prior-Period Claim History eTable 5. Ratio of Actual to Predicted Future Claims eTable 6. Risk Ratios for Future Claims, for Varying Periods eTable 7. Paid Claim Risk by Specialty (Risk Level from Jena et al, 2011) eTable 8. Risk for Future Claims for Illinois high-risk specialties eTable 9. Risk for Future Claims for Illinois Lower-Risk Specialties eTable 10. Effect of Public Disclosure of Paid Claims on Future Claim Risk eFigure 1. Total Paid Claims per 1000 Active Physicians from 1992-2016 [file jamahealthforum-e225436-s001.pdf]

Supplemental Online Content

Hyman DA, Lerner J, Magid DJ, Black B. Association of past and future paid medical malpractice claims. *JAMA Health Forum*. 2023;4(2):e225436. doi:10.1001/jamahealthforum.2022.5436

eAppendix. Simple Model: Medical Malpractice Claims as an Imperfect Proxy for Negligent Care

Simulation Methodology

eTable 1. Risk of a Paid Claim by State

eTable 2. Specialty-Specific Risk of Paid Claims: Illinois Data

eTable 3. Panel A. NPDB Summary statistics by claim year

eTable 3. Panel B. Percentage of Physicians with Paid Claims Over Different Time Periods

eTable 4. Summary statistics, and risk for future-period claims, given prior-period claim history

eTable 5. Ratio of Actual to Predicted Future Claims

eTable 6. Risk ratios for future claims, for varying periods

eTable 7. Paid Claim Risk by Specialty (Risk Level from Jena et al, 2011)

eTable 8. Risk for future claims for Illinois high-risk specialties

eTable 9. Risk for future claims for Illinois lower-risk specialties

eTable 10. Effect of Public Disclosure of Paid Claims on Future Claim Risk

eFigure 1. Total Paid Claims per 1000 Active Physicians from 1992-2016

This supplemental material has been provided by the authors to give readers additional information about their work.

Simple Model: Medical Malpractice Claims as an Imperfect Proxy for Negligent Care

This study treats paid medical malpractice claims as a proxy for medical care that causes patient harm. It is known that only a small percentage of medical errors lead to claims (Baker, 2005), and that not all paid claims involve negligence, but there is also a reasonably high likelihood that a paid claim reflects actual negligence (Studdert et. al, 2006). Imagine a physician, who has skill level *q* (for quality), sees many patients *s* and provides negligent care to a fraction *r(q)* of those patients. However, only a small fraction *v* of negligent care results in a paid claim. Conversely, some fraction *w* of non-negligent care leads to an adverse patient outcome and eventually to a paid claim. The likelihood of a paid claim for physician *i*, in specialty *j* and state s, is:

$$p_{ijs} \sim s_{ij}*[r(q_{ij})*v_{ij})) + (1\text{-}r(q_{ij}))*w_{ij}] + \varepsilon_{ijs} \qquad (1)$$

Here ε_{ijs} is a mean-zero "error" that captures both random noise and other factors that affect paid claim likelihood. In this model, the positive predictive value (the expected fraction of paid claims that are true positives (i.e., that reflect actual negligence)) will be:

$$PPV_{ijs} = \frac{r_{ijs} \times v_{ijs}}{(\mathrm{r_{ijs}} \times v_{ijs}) + (1 - r_{ijs}) \times w_{ijs}} \qquad (2)$$

For low-skill physicians, *v* will be large, most claims will be true positives, and PPV will be high. For high-skill physicians, *v* will be low, PPV will be lower and more claims will be false positives, with no underlying negligence. This can be seen as a simple application of Bayes' theorem: the lower the prior probability of negligence, the lower the posterior probability that an observed paid claim reflects actual negligence.

While the model focuses on paid claims, this tendency will be stronger for all claims, many of which will be closed without payment, than for paid claims. If most physicians are high-skill, this could help explain the common physician belief that most med mal claims are false positives. This

© 2023 Hyman DA et al. *JAMA Health Forum.*

belief could be true for most *physicians*, yet false for most paid *claims*. While we have data only for paid claims, it is possible that the fraction of claims brought which close without payment is higher for high-skill physicians, who may rely on this experience to conclude that claims against other physicians lack a strong basis. A similar dynamic may affect high-skill physicians who are involved in a medical malpractice case where there are also claims against low-skill physicians. A high-skill physician, who is exonerated or dropped from the case may conclude the case lacked merit, even if there was a subsequent payment on behalf of the low-skill physician.[1]

Moreover, high skill physicians may cluster in particular hospitals and geographic areas [2] Thus, high-skill physicians spend most of their time around other high-skill physicians, which could reinforce their experience-based belief that most med mal claims reflect bad luck, rather than bad care.

Note that some low-skill physicians will be "lucky" and not experience claims in the prior period, but will still be more likely to experience future period claims, while some high-skilled physicians will have been unlucky in the prior period and faced a paid claim, but will remain unlikely to face a claim in the future period. Thus, this study of future risk faced by *apparently* skilled versus less-skilled physicians (both defined based on prior period claims) will understate the true effect of physician quality on med mal risk.

The model can be extended by adding a time dimension. In each year, each physician either will or will not experience a paid claim. If the physician does not experience a paid claim, the best estimate of the likelihood of negligent care $r(q)$ will go down; if the physician experiences a paid claim, this estimate will go up. The more prior paid claims, and the more recent they are, the higher the PPV will be for the most recent paid claim.

© 2023 Hyman DA et al. *JAMA Health Forum.*

Simulation Methodology

The primary simulation methodology uses a 5-year period to measure past claims and a 5-year period to measure future claims, with 2009-2013 as the prior period and 2014-2018 as the future period. Note that we compute average claim risk across a number of years, during a time period when the number of claims was dropping steadily (see eFigure 1). Allowing claim risk to vary by year would produce slightly different counts. The approach is similar for other prior and future periods.

The average annual state-specific risk of a paid medical malpractice claim is defined as:

$$f_s = claims_s/docs_s$$

Here $claims_s$ is the average number of claims in state s over 2009-2018, and $docs_s$ is the average number of physicians in state s over 2014-2016 (first 3 years of the "future" period).

For each year in the future period, the study takes $docs_s$ draws of claims from a binomial distribution, with probability f_s for each draw. This produces a number $future_{st}$ of paid claims for each state s and future year t, where $future_{st}$ is a random variable with expected mean = $claims_s$, but the actual values will vary across simulations and future years. For each future state*year, the study randomly assigns each of these claims to one of the physicians in that state. The study then counts the number of suits for each physician for each future year, and sums these physician-specific counts over the future period. Since f_s is small (the national average is around 1%), most physician*years will have zero claims, a few (approximately f_s) will have one claim, a smaller number (approximately f_s^2) will have two claims, and so on.

If claims arrived at random, the likelihood that a physician would receive one claim in 5 future years will be about $5*f_s$ (about 5% for an average state), the likelihood of two claims will be about $(5*f_s)^2$, or around 0.25%, and so on. (These back-of-the-envelope estimates are reasonably close to the

© 2023 Hyman DA et al. *JAMA Health Forum.*

actual values from Table 1, simulation row, which shows a 4.78% likelihood that a physician will receive 1+ future claims in the next 5 years and an 0.12% likelihood of 2+ future claims.) We run each simulation 10,000 times, and thus obtain a mean number of physicians who will receive 1 paid claim (say) in the future period, and a distribution around that mean. We use this distribution to determine the 95% confidence interval around the mean number.

We also use NPDB data to determine the actual number of physicians who receive 1, 2, 3, etc. paid claims during the prior period and the future period.

eTable 1. Risk of a Paid Claim by State

Table shows the risk that physicians in a particular state will have a paid med mal claim, state-specific risk relative to national average, overall risk rank, and whether the state has public disclosure of paid claims. Paid claims are annual average by state over 2011-2015, rounded to nearest whole number, using the licnstat variable in NPDB. Active physician counts are based on data from AHRF, averaged over the same time period. States are sorted by risk level, from highest to lowest.

	(1)	(2)	(3) = 1000*[(1)/(2)]	(4) = (3)/nat'l avg		
**State**	**Paid claims**	**Active physicians**	**Claims/1,000 physicians**	**Risk relative to national avg**	**Risk rank**	**Med Mal Disclosure**
West Virginia	91	4,219	21.6	2.422	1	Yes
New York	1,352	77,822	17.4	1.950	2	Yes
Montana	35	2,108	16.4	1.838	3	No
Oklahoma	93	6,642	14	1.572	4	No
New Jersey	384	27,452	14	1.568	5	Yes
Mississippi	73	5,336	13.7	1.536	6	No
Florida	653	48,242	13.5	1.519	7	Yes
New Mexico	66	4,908	13.5	1.516	8	No
Louisiana	166	12,679	13.1	1.472	9	No
Pennsylvania	482	39,566	12.2	1.366	10	No
Indiana	172	14,327	12	1.346	11	Yes
Utah	70	6,165	11.3	1.270	12	No
Wyoming	11	1,007	10.9	1.227	13	No
New Hampshire	42	3,873	10.9	1.225	14	Yes
Kansas	69	6,493	10.6	1.193	15	No
Kentucky	107	10,158	10.5	1.180	16	No
Maryland	244	23,394	10.4	1.172	17	Yes
Rhode Island	42	4,083	10.3	1.155	18	No
Nevada	51	5,034	10.1	1.132	19	No
Arizona	145	14,695	9.9	1.108	20	No
Arkansas	56	6,034	9.3	1.042	21	No
Delaware	21	2,256	9.2	1.033	22	No
Michigan	235	26,479	8.9	0.994	23	No
South Carolina	96	10,911	8.8	0.983	24	No

© 2023 Hyman DA et al. *JAMA Health Forum.*

	(1)	(2)	(3) = 1000*[(1)/(2)]	(4) = (3)/nat'l avg		
**State**	**Paid claims**	**Active physicians**	**Claims/1,000 physicians**	**Risk relative to national avg**	**Risk rank**	**Med Mal Disclosure**
Maine	31	3,635	8.5	0.957	25	No
California	863	103,632	8.3	0.935	26	Yes
Iowa	48	5,810	8.3	0.928	27	No
Idaho	22	2,646	8.2	0.923	28	No
Georgia	179	22,133	8.1	0.909	29	Yes
Illinois	288	37,693	7.6	0.858	30	Yes
Missouri	116	15,388	7.5	0.846	31	Yes
Nebraska	34	4,647	7.3	0.815	32	No
Connecticut	98	13,724	7.1	0.802	33	Yes
South Dakota	13	1,861	7.1	0.799	34	No
Washington	127	18,475	6.9	0.769	35	No
Texas	389	57,683	6.7	0.757	36	No
Alaska	10	1,547	6.6	0.744	37	No
Oregon	74	11,152	6.6	0.743	38	Yes
Massachusetts	209	32,723	6.4	0.716	39	Yes
Tennessee	107	17,213	6.2	0.700	40	Yes
Virginia	132	21,687	6.1	0.683	41	Yes
Colorado	79	13,925	5.6	0.633	42	Yes
Ohio	160	32,700	4.9	0.550	43	No
North Carolina	117	25,026	4.7	0.524	44	Yes
Alabama	44	10,441	4.2	0.470	45	No
Hawaii	15	4,010	3.7	0.420	46	No
Vermont	9	2,333	3.6	0.409	47	Yes
North Dakota	6	1,728	3.5	0.39	48	No
Wisconsin	44	14,942	2.9	0.327	49	No
District of Columbia	13	4,955	2.7	0.300	50	No
Minnesota	40	16,455	2.5	0.275	51	No
**All physicians**	8,315	863,767	8.9	1.000		
**States w. med mal data avail.**	5,209	525,958	9.3	1.050		
**States w/o med mal data avail.**	2,814	346,089	8.6	0.971		

© 2023 Hyman DA et al. *JAMA Health Forum.*

eTable 2: Specialty-Specific Risk of Paid Claims: Illinois Data

Number of licensed Illinois physicians in indicated specialties and surgical sub-specialties, and paid claim risk over 1990-2016, relative to the average risk for all physicians with known specialty. Relative to the AHRF specialties shown in eTable 2, the Illinois data does not include plastic surgery (included in general surgery in Illinois); gastroenterology, cardiology, and pulmonary medicine (all included in internal medicine in Illinois), and radiation and oncology (included in radiology in Illinois).

**AHRF specialty**	**Illinois Specialty**	**No. of physicians**	**Relative risk**
General Surgery	General Surgery	1,726	2.49
Orthopedic Surgery	Orthopedic surgery*	1,349	2.84
Cardiovascular surgery	Cardio-thoracic surgery	409	1.88
Neurosurgery	Neurosurgery	341	3.33
N/A	Vascular surgery	147	1.16
N/A	Colorectal surgery	76	1.42
**All Surgeons**	All surgery	4,048	2.56
Obstetrics and Gynecology	Ob-gyn*	2,330	2.70
Urology	Urology*	537	1.33
**All high-risk specialties**		**6,915**	2.5
Diagnostic Radiology	Radiology	4,056	0.93
Anesthesiology	Anesthesiology	2,548	0.56
Emergency Medicine	Emergency medicine*	2,326	0.95
Neurology	Neurology	1,002	0.58
Internal Medicine	Internal medicine	13,357	0.67
Pathology	Pathology	1,639	0.42
Ophthalmology	Ophthalmology	1,102	0.81
Dermatology	Dermatology	621	0.53
Family General Practice	Family physicians	5,335	0.86
	Physical medicine	584	0.16
Other Specialties	Other specialties	223	0.21
Pediatrics	Pediatrics	4,130	0.51
Psychiatry	Psychiatry	1,969	0.35
**All Illinois Physicians (with known specialty)**		**46,890**	**1.00**
Other physicians	Unknown specialty	41,712	1.12
**All active physicians**	**All physicians**	**88,602**	**1.02**

© 2023 Hyman DA et al. *JAMA Health Forum.*

eTable 3, Panel A. NPDB Summary statistics by claim year

Panel A provides summary statistics for NPDB claims paid by physicians (MDs) by claim year, and for indicated periods. For multiyear ranges, % of physicians with paid claims is based on average number of active physicians during the period. Physician counts for 2014-2015 are extrapolated based on trends over 2011-2013.

	(1)	(2)	(3)	(4) = (1)/(3)	(5) = (2)/(3)
**Year or range**	**Paid claims**	**Distinct physicians with paid claims**	**Active Physicians**	**Claims per Active Physician**	**Percentage of Physicians with 1+ paid claims**
1992	13,340	12,198	568,132	0.0235	2.15%
1993	13,278	12,119	576,771	0.0230	2.10%
1994	13,775	12,674	589,906	0.0234	2.15%
1995	12,635	11,687	617,362	0.0205	1.89%
1996	13,137	12,076	634,775	0.0207	1.90%
1997	12,970	11,733	656,195	0.0198	1.79%
1998	12,454	11,302	678,649	0.0184	1.67%
1999	13,295	12,277	693,345	0.0192	1.77%
2000	13,699	12,709	708,463	0.0193	1.79%
2001	14,625	12,461	720,933	0.0203	1.73%
2002	13,527	12,462	737,495	0.0183	1.69%
2003	13,331	12,372	755,287	0.0177	1.64%
2004	12,658	11,745	760,751	0.0166	1.54%
2005	12,233	11,158	769,730	0.0159	1.45%
2006	10,921	10,158	778,331	0.0140	1.31%
2007	9,986	9,255	794,184	0.0126	1.17%
2008	9,702	9,013	797,864	0.0122	1.13%
2009	9,420	8,794	810,218	0.0116	1.09%
2010	8,832	8,259	822,571	0.0107	1.00%
2011	8,680	7,994	833,668	0.0104	0.96%
2012	8,225	7,642	847,977	0.0097	0.90%
2013	8,242	7,522	862,444	0.0096	0.87%
2014	8,111	7,624	875,994	0.0093	0.87%
2015	7,882	7,437	898,750	0.0088	0.83%
2016	7,405	7,012	915,368	0.0081	0.77%
2006-2010	48,861	40,938	800,634	0.0611	5.11%
2011-2015	41,140	34,725	863,767	0.0477	4.02%
2006-2015	90,001	69,729	832,200	0.1088	8.38%
1996-2005	131,929	94,430	711,562	0.1861	13.27%
1996-2015	221,930	145,915	771,881	0.2950	18.90%
1992-2016	282,363	175,886	748,207	0.3934	23.51%

© 2023 Hyman DA et al. *JAMA Health Forum.*

eTable 3, Panel B. Percentage of Physicians with Paid Claims Over Different Time Periods

Panel B shows percentage of active, practicing non-federal physicians with indicated numbers of paid claims, over indicated periods. Number of physicians is averaged over indicated period.

Year Range	No of years	1+ Claims	2+ Claims	3+ Claims	4+ Claims
2006-2010	5	5.11%	0.70%	0.16%	0.05%
2011-2015	5	4.02%	0.50%	0.11%	0.03%
2006-2015	10	8.38%	1.57%	0.44%	0.17%
1996-2005	10	13.27%	3.21%	1.02%	0.41%
2001-2015	15	13.88%	3.38%	1.12%	0.46%
1996-2015	20	20.51%	5.95%	2.21%	0.99%
1992-2016	25	23.51%	7.52%	3.03%	1.42%

© 2023 Hyman DA et al. *JAMA Health Forum.*

eTable 4. Summary statistics, and risk for future-period claims, given prior-period claim history

Panel A is similar to text Table 1 and shows the number of active, practicing non-federal physicians with indicated numbers of paid medical malpractice claims reported to the NPDB during the baseline period (2009-2013) (left portion) and paid claims reported to the NPDB during the future period (2014-2018). Difference from Table 1 is that in this table we report separately data for physicians with 3 versus 4+ paid claims during the baseline and future periods. **Panel B** shows the predicted average **probability** across all states for a physician to have the indicated numbers of paid claims during the future period, if claims arrive at the average state-specific rates observed for the baseline period, but otherwise at random (independent of physician skill) and all physicians active during the baseline period remain active in the future period. Probabilities are mean values from 10,000 simulations, averaged across states, with states weighted by average number of active physicians in the baseline period. **Panel C** shows the predicted **numbers** of physicians with the indicated numbers of paid claims during the future period under the same assumptions as Panel B. Predicted numbers are mean values from 10,000 simulations. **Panel D** shows ratio of actual to predicted claims during the future period.

Panel A. Actual numbers of physicians with indicated numbers of baseline and future claims

**Baseline period (2009-2013)**					**Future period (2014-2018)**					
**Baseline period paid claims**	**Physicians**		**Total claims**		**Number of physicians with**					
	**Number**	**%**		**0 paid claims**	**1 paid claim**	**2 paid claims**	**3 paid claims**	**4+ paid claims**	**1 or more claims**	**% with 1+ claims**
0	841,961	95.92%	0	814,036	25,051	2,370	363	141	27,925	3.32%
1	34,512	3.42%	34512	30,236	3,466	604	143	63	4,276	12.39%
2	4,189	0.51%	8378	3250	669	189	48	33	939	22.42%
3	800	0.10%	2400	523	185	48	28	16	277	34.63%
4+	414	0.05%	2269	242	89	35	20	28	172	41.55%
**Total**	**881,876**	**100%**	**47,559**	**848,287**	**29,460**	**3,246**	**602**	**281**	**33,589**	3.81%
**Total actual future claims**					**39,691**					

Panel B. Predicted probability for physician to have indicated numbers of future claims, if claims arrive at random

	**Number of paid claims during future period**					
	**0**	**1**	**2**	**3**	**4+**	**1 or more**
Probability	95.33%	4.56%	0.11%	0.002%	0.00002%	4.67%

Panel C. Predicted number of physicians with indicated numbers of future claims, if claims arrive at random

**Baseline period paid claims**	**No. of Physicians**	**Predicted number of physicians with**					
		**0 paid claims**	**1 paid claim**	**2 paid claims**	**3 paid claims**	**4+ paid claims**	**1 or more claims**
0	841,961	802,622	38,390	920	15	0.19	39,324
1	34,512	32,899	1574	38	0.60	0.008	1,612
2	4,189	3,993	191	4.6	0.07	0.0009	196
3	800	762	36	0.87	0.014	0.0002	37
4+	414	395	19	0.45	0.007	0.00009	19
**Total**	**881,876**	840,672	40,209	963.32	15.38	0.20	41,188
**Total predicted future claims**			**42,186**				

Panel D. Ratio: Actual to Predicted Future Claims

**Baseline period paid claims**	**No. of Physicians**	**Ratio of Actual/Predicted number of physicians with**					
		**0 paid claims**	**1 paid claim**	**2 paid claims**	**3 paid claims**	**4+ paid claims**	**1 or more claims**
0	841,961	1.014	0.653	2.58	24.7	743	0.71
1	34,512	0.919	2.20	16.02	238	8,096	2.65
2	4,189	0.814	3.50	41.3	657	34,938	4.79
3	800	0.686	5.07	54.9	2,006	88,700	7.41
4+	414	0.613	4.72	77.4	2,769	299,951	8.89
**Total**	**881,876**	1.01	0.73	3.37	39	1,413	**0.82**

© 2023 Hyman DA et al. *JAMA Health Forum.*

eTable 5. Ratio of Actual to Predicted Future Claims

Table shows ratios of actual to predicted numbers of future-period paid claims, for physicians with indicated numbers of prior-period paid claims. These ratios are presented graphically in text Figure 1.

**No. of paid claims (baseline period)**	**No. of Physicians**	**Ratio of Actual/Predicted number of physicians**				
		**0 paid claims**	**1 paid claim**	**2 paid claims**	**3+ paid claims**	**1+ paid claims**
0	841,961	1.0	0.7	2.6	34	0.71
1	34,512	0.9	2.2	16	338	2.65
2	4,189	0.8	3.5	41	1,094	4.79
3+	800	0.7	4.95	63	6,506	8.02
**Total**	**881,876**	**1.009**	**0.73**	**3.37**	**39.1**	**0.82**

© 2023 Hyman DA et al. *JAMA Health Forum.*

eTable 6: Risk ratios for future claims, for varying periods

Table parallels text Table 2, and shows the ratio of the risk for distribution of 1+ (Panel A), 2+ (Panel B) and 3+ (Panel C) paid claims over varying future periods among active practicing physicians based on each physician's number of paid claims over the baseline period), compared to the likelihood assuming claims arrive randomly at the average rate during the baseline period. Base probabilities with random claim arrival assume that all future period claims involve physicians active during the baseline period, and are mean values from 1,000 simulations, assuming claims arrive randomly at state-specific rates. Ratio for physicians with 0 baseline claims for having 1+ future claims is less than 1.00 due to lower risk for these physicians plus secular decline in paid claim rate, but we normalize this ratio to 1.00 for each specified future period to simplify interpretation.

Panel A: 1+ Future Claims

**Future period (years)**	**1**	**2**	**3**	**4**	**5**	**6**	**7**	**8**	**9**
Future period	2018	2017-18	2016-18	2015-18	2014-18	2013-18	2012-18	2011-18	2010-18
Baseline period	2013-17	2012-16	2011-15	2010-14	2009-13	2008-12	2007-11	2006-10	2005-09
**Baseline period paid claims**									
0	1.00	1.00	1.00	1.00	1.00	1.00	1.00	1.00	1.00
1	4.43	4.60	4.23	3.78	4.42	3.95	3.76	3.50	3.35
2	8.16	8.78	7.81	6.84	7.07	7.20	6.71	6.07	5.53
3	13.64	15.10	11.90	10.54	11.26	9.24	9.79	8.66	8.09
4+	27.16	22.02	13.76	12.95	15.16	15.12	11.98	10.38	10.41
**Base probability (random claim arrival)**	1.73%	2.27%	3.43%	4.67%	4.51%	5.19%	6.10%	7.11%	8.11%

Panel B: 2+ future claims

**Future period (years)**	**1**	**2**	**3**	**4**	**5**	**6**	**7**	**8**	**9**
Future period	2018	2017-18	2016-18	2015-18	2014-18	2013-18	2012-18	2011-18	2010-18
baseline period	2013-17	2012-16	2011-15	2010-14	2009-13	2008-12	2007-11	2006-10	2005-09
**Baseline period paid claims**									
0	1.00	1.00	1.00	1.00	1.00	1.00	1.00	1.00	1.00
1	8.75	7.86	5.96	6.24	7.33	6.80	6.38	6.00	8.75
2	27.36	23.64	16.90	16.74	20.85	17.40	16.53	14.82	27.36
3	68.99	56.86	42.38	36.31	36.98	36.36	27.57	27.62	68.99
4+	114.66	87.21	51.06	52.65	62.33	60.12	40.38	41.69	114.66
**Base probability (random claim arrival)**	0.015%	0.018%	0.069%	0.091%	0.104%	0.137%	0.190%	0.259%	0.337%

© 2023 Hyman DA et al. *JAMA Health Forum.*

Panel C: 3+ Future Claims

**Future period (years)**	**1**	**2**	**3**	**4**	**5**	**6**	**7**	**8**	**9**
Future period	2018	2017-18	2016-18	2015-18	2014-18	2013-18	2012-18	2011-18	2010-18
baseline period	2013-17	2012-16	2011-15	2010-14	2009-13	2008-12	2007-11	2006-10	2005-09
**Baseline period paid claims**									
0	1.00	1.00	1.00	1.00	1.00	1.00	1.00	1.00	1.00
1	12.21	12.18	13.15	12.76	12.10	11.70	11.00	10.00	9.73
2	40.79	40.65	50.29	55.15	39.14	44.57	33.16	30.29	28.40
3	194.02	193.31	143.78	133.97	105.72	94.48	98.32	69.71	64.93
4+	983.31	435.42	424.17	395.58	318.62	249.00	313.16	185.06	133.87
**Base probability (random claim arrival)**	0.00008%	0.0002%	0.0006%	0.0009%	0.001%	0.002%	0.004%	0.006%	0.009%

© 2023 Hyman DA et al. *JAMA Health Forum.*

eTable 7. Paid Claim Risk by Specialty (Risk Level from Jena et al., 2011)

Table shows the national annual risk that physicians in the indicated AHRF specialties will have a paid med mal claim, relative to the average for all specialties, adapted from Jena et al. (2011). We do not use the Jena et al. category of "other specialties" and instead compute the annual and relative risks for "other physicians" (not in any of the named specialties) to ensure that weighted average relative risk for all physicians = 1. Annual risks are average over 2011-2015. Physician counts are based on data from AHRF, averaged over 2011-2015. Specialties are listed in decreasing risk order. Annual specialty risk = average annual ratio of claims/physician for all active physicians over 2011-2015 (from Table 1) * relative risk. "All surgeons" is weighted average of other surgeon rows. 5-year risk is simulated assuming random arrival of claims.

AHRF specialty	Jena Specialty	No. of physicians	Relative risk	Annual risk (1+ claims)	5-year risk (1+ claims)
General Surgery	General Surgery	37,510	2.62	2.49%	13.46%
Orthopedic Surgery	Orthopedic Surgery	25,603	2.44	2.32%	12.63%
Cardiovascular surgery	Thoracic-cardiovasc Surgery	4,509	2.42	2.30%	13.32%
Neurosurgery	Neurosurgery	6,138	1.94	1.84%	10.11%
Plastic Surgery	Plastic Surgery	7,775	1.74	1.65%	9.30%
**All Surgeons**		**81,535**	**2.42**	**2.30%**	**13.26%**
Obstetrics and Gynecology	Obstetrics and Gynecology	38,662	1.82	1.73%	9.51%
Urology	Urology	10,583	1.57	1.49%	7.82%
Radiology and Oncology	Oncology	5,030	1.18	1.12%	6.36%
Diagnostic Radiology	Diagnostic Radiology	25,960	1.02	0.97%	5.62%
Anesthesiology	Anesthesiology	44,569	1.01	0.96%	5.44%
Emergency Medicine	Emergency Medicine	36,828	0.91	0.86%	4.99%
Neurology	Neurology	17,069	0.89	0.85%	5.05%
Gastroenterology	Gastroenterology	13,756	0.84	0.80%	4.64%
Internal Medicine	Internal Medicine	11,5223	0.81	0.77%	4.40%
Pathology	Pathology	1,8214	0.81	0.77%	4.56%
Ophthalmology	Ophthalmology	18,651	0.74	0.70%	3.96%
Dermatology	Dermatology	11,723	0.73	0.69%	3.64%
Family General Practice	Family General Practice	87,718	0.65	0.62%	3.51%
Cardiology	Cardiology	22,620	0.60	0.57%	3.26%
Pulmonary Medicine	Pulmonary Medicine	11,877	0.59	0.56%	2.75%
Other Specialties	Other Specialties	5,698	0.45	0.43%	2.42%
Pediatrics	Pediatrics	58,167	0.33	0.31%	1.83%
Psychiatry	Psychiatry	37,651	0.28	0.27%	1.41%
Other physicians		202,232	0.997	0.95%	6.09%
**All active physicians**	**All physicians**	**863,767**	**1.000**	**0.95%**	**6.09%**

© 2023 Hyman DA et al. *JAMA Health Forum.*

eTable 8. Risk for future claims for Illinois high-risk specialties

Sample is med mal claims paid by Illinois physicians in high-risk specialties over 1990-2016, as reported to IDFPR. High-risk specialties are defined as obstetrics and gynecology, surgery (including all surgery subspecialties), urology, and otolaryngology. **Panel A** shows number of Illinois physicians with indicated numbers of baseline paid medical malpractice claims reported to the during 2005-2009 (left portion) and "future" paid claims during 2010-2014. Total number of physicians is averaged over 2003-2007. **Panel B** shows predicted number of future paid claims if claims arrive randomly at the average rate for all high-risk specialties for the baseline period and involve physicians active during the baseline period. Predicted numbers are mean values from 10,000 simulations. **Panel C**. Ratio of actual future claims (from Panel A) to predicted future claims (from Panel B).

Panel A. High-risk Illinois physicians with indicated numbers of baseline and future claims: actual

Baseline period (2005-2009)				Future period (2010-2014)				
Baseline period paid claims	Physicians		Total claims	Number of physicians with				
	Numbers	%		0 paid claims	1 paid claim	2+ paid claims	1 or more claims	% with 1+ claims
0; also 0 before 2005	5,493	83%	0	5,272	203	18	221	4%
0, and 1+ during 1990-2004	1058	16%	0	69	69	8	77	7%
0	6,586	93%	0	6,285	274	271	301	4.6%
1	421	6%	421	362	52	7	59	14.0%
2+	56	1%	124	47	7	2	9	16.1%
**Total**	**7,063**	**100%**	**545**	**6,694**	**333**	**36**	**369**	5.2%

Panel B. Predicted number of Illinois physicians with indicated numbers of future claims, if claims arrive at random

Baseline period paid claims	No. of Physicians	Predicted number of physicians with:			
		0 paid claims	1 paid claim	2+ paid claims	1+ claims
0	6,586	5913	636	35.7	672
1	421	378	40.7	2.21	42.9
2+	56	50	5.41	0.05	5.46
**Total**	**7,063**	**6341**	**682**	**38.3**	**720**
	**Total Predicted future claims**				

Panel C. Ratio: Actual to Predicted Future Claims for High-Risk Physicians

Baseline period paid claims	No. of Physicians	Ratio: Actual/Predicted number of physicians with				
		0 paid claims	1 paid claim	2+ paid claims	1+ claims	1+ claim vs. 0 baseline
0	6,586	1.06	0.43	0.76	0.45	1
1	421	0.96	1.28	3.16	1.38	3.1
2+	56	0.93	1.29	42.0	1.65	3.7
**Total**	**7,063**	1.056	0.49	0.94	0.51	1.13

© 2023 Hyman DA et al. *JAMA Health Forum.*

eTable 9: Risk for future claims for Illinois lower-risk specialties

Table format is similar to eTable 8, except sample is med mal claims for Illinois physicians in lower-risk specialties (all listed specialties, other than the high-risk specialties included in eTable 8).

Panel A. Lower-risk Illinois physicians with indicated numbers of baseline and future claims: actual

Baseline period (2005-2009)				Future period (2010-2014)				
Baseline period paid claims	Physicians		Total claims	Number of physicians with				
	Numbers	%		0 paid claims	1 paid claim	2+ paid claims	1+ claims	% with 1+ claims
0; also 0 before 2005	31,575	95%	0	31,112	451	12	463	1.5%
0, and 1+ during 1990-2004	1,811	5%	0	1,719	88	4	92	5.1%
0	33,390	98%	0	32,835	539	16	555	1.7%
1	732	2%	732	682	47	3	50	6.8%
2+	35	0%	73	32	3	0	3	8.6%
**Total**	**34,157**	**100%**	**805**	**33,549**	**589**	**19**	**608**	**1.8%**

Panel B. Predicted number of Illinois physicians with indicated numbers of future claims, if claims arrive at random

Baseline period paid claims	No. of Physicians	Predicted number of physicians with:			
		0 paid claims	1 paid claim	2+ paid claims	1+ claims
0	33,390	32,267	1103	19.0	1,122
1	732	707	24.2	0.22	24.4
2+	35	33.8	1.16	0.02	1.18
**Total**	**34,157**	**33,009**	**1,129**	**19.46**	**1,148**
	**Total Predicted future claims**				

Panel C. Ratio: Actual to Predicted Future Claims for Lower-Risk Physicians

Baseline period paid claims	No. of Physicians	Ratio of Actual/Predicted number of physicians with				
		0 paid claims	1 paid claim	2+ paid claims	1+ claims	1+ claim vs. 0 baseline
0	33,390	1.02	0.49	0.84	0.49	1
1	732	0.96	1.94	13.9	2.05	4.2
2+	35	0.95	2.59	0	2.55	5.2
**Total**	**34,157**	1.02	0.52	0.98	0.53	1.1

© 2023 Hyman DA et al. *JAMA Health Forum.*

eTable 10. Effect of Public Disclosure of Paid Claims on Future Claim Risk

Table provides numerical values shown graphically in text Figure 2, and shows likelihood, relative to physicians with zero baseline period paid claims, for physicians with either one or two paid claims in the baseline five years, for each of the next five years, separately for states with and without public reporting of paid claims. **, *** indicates statistical significance at the 5% or 1% level. Statistically significant results shown in **boldface**.

	**Claim Likelihood (relative to physicians with 0 baseline claims)**					
Paid Claims in Last Five Years	**1 Claim**			**2 Claims**		
Past Paid Claims are	Public	Not Public	Diff.	Public	Not Public	Diff.
Future Year:						
1	5.12	5.13	-0.01	11.81	13.73	**-1.92*** **
2	4.67	4.45	0.22	10.48	10.52	-0.04
3	4.25	3.98	0.27	9.31	9.28	0.03
4	3.95	3.74	0.21	8.38	8.43	-0.05
5	3.78	3.5	0.28	7.65	7.51	0.14

© 2023 Hyman DA et al. *JAMA Health Forum.*

eFigure 1: Total Paid Claims per 1000 Active Physicians from 1992-2016

Paid med mal claims reported per NPDB, per 1,000 active practicing non-federal physicians.

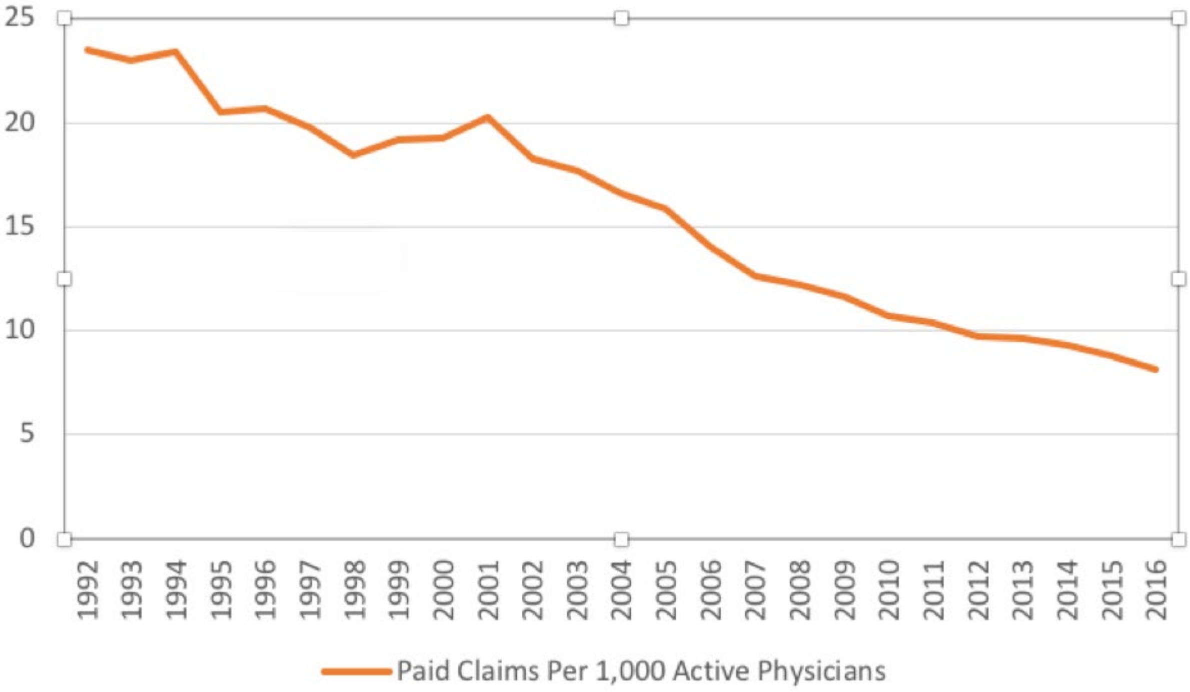

---

[1] Rahmati, M, Hyman DA, Black B, Liu J, Silver C. Screening Plaintiffs and Selecting Defendants in Medical Malpractice Litigation: Evidence from Illinois and Indiana, J. Empirical Legal Studies, 2018; 15: 41-79.

[2] Hyman DA, Rahmati M, Black B. Medical Malpractice and Physician Discipline: The Good, The Bad, and The Ugly. J. Empirical Legal Studies, 2021; 18:131-166.

© 2023 Hyman DA et al. *JAMA Health Forum.*
