# Supplementary material for: Association of Past and Future Paid Medical Malpractice Claims
Source: JAMA Health Forum. 2023 Feb 10;4(2):e225436. doi: 10.1001/jamahealthforum.2022.5436 (PMC9918873; doi:10.1001/jamahealthforum.2022.5436)
Supplement: Supplement 2. — Data Sharing Statement [file jamahealthforum-e225436-s002.pdf]

Data Sharing Statement

Hyman. Association of Past and Future Paid Medical Malpractice Claims. *JAMA Health Forum*. Published February 10, 2023. doi:10.1001/jamahealthforum.2022.5436

Data
Data available: Yes
Data types: Deidentified participant data
How to access data: bblack@northwestern.edu
When available: With publication

Supporting Documents
Document types: None

Additional Information
Who can access the data: Researchers whose proposed use of the data has been approved
Types of analyses: Any research purpose
Mechanisms of data availability: With a signed data access agreement
